# Poly(Acrylic acid)–Based Hybrid Inorganic–Organic Electrolytes Membrane for Electrical Double Layer Capacitors Application

**DOI:** 10.3390/polym8050179

**Published:** 2016-05-18

**Authors:** Chiam-Wen Liew, H.M. Ng, Arshid Numan, S. Ramesh

**Affiliations:** 1 Centre for Ionics University of Malaya, Department of Physics, Faculty of Science, University of Malaya, LembahPantai, 50603 Kuala Lumpur, Malaysia; liewchiamwen85@gmail.com (C.-W.L.); nghonming@hotmail.com (H.M.N.); numan.arshed@yahoo.com (A.N.); 2 School of Foundation Studies, Xiamen University Malaysia Campus, Jalan Suria Serenia 1, Bandar Serenia, 43900 Sepang, Malaysia

**Keywords:** poly(acrylic acid), TiO_2_, nanocomposite polymer electrolyte membrane, EDLCs, capacitance

## Abstract

Nanocomposite polymer electrolyte membranes (NCPEMs) based on poly(acrylic acid)(PAA) and titania (TiO_2_) are prepared by a solution casting technique. The ionic conductivity of NCPEMs increases with the weight ratio of TiO_2_.The highest ionic conductivity of (8.36 ± 0.01) × 10^−4^ S·cm^−1^ is obtained with addition of 6 wt % of TiO_2_ at ambient temperature. The complexation between PAA, LiTFSI and TiO_2_ is discussed in Attenuated total reflectance-Fourier Transform Infrared (ATR-FTIR) studies. Electrical double layer capacitors (EDLCs) are fabricated using the filler-free polymer electrolyte or the most conducting NCPEM and carbon-based electrodes. The electrochemical performances of fabricated EDLCs are studied through cyclic voltammetry (CV) and galvanostatic charge-discharge studies. EDLC comprising NCPEM shows the specific capacitance of 28.56 F·g^−1^ (or equivalent to 29.54 mF·cm^−2^) with excellent electrochemical stability.

## 1. Introduction

Solid polymer electrolyte (SPE) membranes are good candidates to replace conventional liquid electrolytes because of their attractive properties, such as ease of handling and manufacturing, the fact they can be configured in any shape due to their flexible polymer matrix, their wide operating temperature range, low volatility, high energy density, no new technology requirements, low electronic conductivity and negligible vapor pressure [1,2,3,4]. Other features are excellent electrochemical, structural, thermal, photochemical and chemical stabilities, lightness in weight and better safety performance, as SPEs can eliminate the problems of corrosive solvent leakage and harmful gas during operation [5,6,7]. However, the ionic conductivity of SPEs is extremely low. Therefore, several approaches have been taken to improve the ionic conductivity of polymer electrolytes; for example, addition of additives like plasticizers, ionic liquids, fillers and/or liquid crystals, blending of two different polymers, modifications on the polymers and irradiation with gamma (γ) rays as well as mix salt systems.

Among all the approaches, doping of fillers is an alternative way to enhance the ionic conductivity of SPEs. Apart from ionic conductivity, addition of fillers increases the mechanical integrity of polymer electrolyte membranes. Addition of fillers can improve interfacial stability between electrodes and electrolytes, enhance cationic diffusivity by altering the ionic transport properties, reduce the water retention of polymer electrolyte membranes and decrease glass transition temperature (*T*_g_) of the polymer matrix [8,9,10,11,12,13]. Nanotechnology has received an upsurge of interest in recent years. Nano-sized fillers have become popular materials to substitute the larger particle size of fillers due to their high surface area. Nano-scaled fillers provide high activity, exhibit good chemical stability and form rapidly in the space charge region between the grains in comparison to micro-sized fillers [8,14,15]. PAA is a non-toxic, hydrophilic and biocompatible superabsorbent polymer with a three dimensional (3-D) network [16,17,18]. PAA was chosen as a host polymer in this research due to its fascinating behaviors such as excellent stability in acidic and basic media, high ionic conductivity, strong adhesive properties, superior selectivity and permeability and high ability to associate with a variety of multivalent metal ions in solution [18,19,20,21]. PAA can suppress the crystallization and form stable complexes with metal [18,22,23]. The main reason for choosing PAA as a host polymer is because of its high charge density based on carboxylic (–COOH) functional group. This functional group favors the bond formation—for example, ionic, covalent, hydrogen and coordination–which can be used to form complexation with the nanoparticles [24,25]. The ionization of the carboxylic group in PAA depends mainly on pH and ionic strength [26,27]. The charge density of PAA is low in acidic media due to the poor degree of dissociation. The ions can just be dissociated well in the solution at higher pH values (pH > 5).

Lithium bis(trifluoromethane)sulfonimide (LiTFSI) was selected because of its non-corrosive behavior towards electrodes, wide electrochemical stability, excellent thermal stability and superior thermal properties. Apart from that, this salt can dissociate very well even in low dielectric solvents. It is a newly designed metal salt to replace the poor conducting lithium triflate (LiTf), the hazardous lithium perchlorate (LiClO_4_), the thermally unstable LiBF_4_ and lithium hexafluorophosphate (LiPF_6_), and the toxic lithium hexafluoroarsenate (LiAsF_6_) [28]. The extensive delocalized electrons in TFSI^−^ anion can promote the ion dissociation and thereby increase the ionic conductivity [29]. The attempt of using TiO_2_ is due to its numerous outstanding features, such as improved physical and electrochemical stabilities, enhanced cationic transport number, hydrophilic, good mechanical integrity, improved sinterability and excellent stability in acidic and oxidative environments with free-flowing structure [14,30,31,32]. Other properties are excellent mechanical and thermal resistances, hydrophilic nature and high ionic exchange capacity (IEC) [10,33]. There are many applications for TiO_2_ such as beam splitters, optical and anti-reflection coatings, heterogeneous catalysis, gas sensors, ultraviolet (UV) absorbers, lithium batteries, optical, electronic and electrochromic devices, self-cleaning surface coatings, UV-resistant coatings and paints, solar cells, disinfectant sprays, water treatment agents and topical sunscreens. Nano-sized TiO_2_ is also used in clean technologies such as environmental remediation, pigments, paints, ceramics, cosmetics and solar energy conversion due to its high photocatalytic activity and chemical stability [30,31,34,35]. Ning and coworkers prepared Mn-doped TiO_2_ micro/nanostructure porous film through an anodization of Ti–Mn alloy. The film annealed at 300 °C produces the highest capacitance of 1451.3 mF/cm^2^ at a current density of 3 mA/cm^2^. This film can be used as a high-performance supercapacitor electrode [36]. TiO_2_ can also be used to improve photocatalytic degradation of methylene blue by preparing reduced graphene-oxide/titanium dioxide/zinc oxide ternary nanocomposites as proven by Raghavan *et al.* [37]. To date, EDLC based on PAA–LiTFSI–TiO_2_ polymer electrolyte membranes have never been reported in literature. Therefore, the main aim of this research work is to investigate the effect of TiO_2_ nanoparticles on PAA–LiTFSI polymer matrix and EDLC application.

## 2. Experimental Section

### 2.1. Materials

The NCPEMs comprising PAA, LiTFSI and TiO_2_ were prepared by a solution casting method. PAA (from Sigma-Aldrich, St. Louis, MI, USA, molecular weight of 3,000,000 g·mol^−1^), LiTFSI (from Sigma-Aldrich) and TiO_2_ (from Sigma-Aldrich with particle size of <100 nm) are used as host polymer, doping salt and fillers, respectively. All the materials were obtained from Sigma-Aldrich. All the materials were used as received.

### 2.2. Preparation of Filler Added Poly(Vinyl Alcohol)-Based Polymer Electrolyte Membranes

PAA–LiTFSI solution was initially dissolved in deionized water. After that, an appropriate amount of TiO_2_ was added into the solution. The resulting solution was subjected to sonicator for 1 h at 70 °C and stirred overnight at 50 °C. The solution was then cast on Petri dish and thus dried in an oven at 50 °C. Polymer electrolyte membranes were eventually formed. The sample designation and the weight ratio of each material used in the research are tabulated in Table 1.

### 2.3. Characterization of Polymer Electrolyte Membranes

#### 2.3.1. Ionic Conductivity Studies

Ionic conductivity of polymer electrolyte membranes was studied by HIOKI 3532-50 LCR HiTESTER impedance analyzer (HIOKI, Nagano, Japan) over a frequency range between 50 Hz and 5 MHz at a signal level of 10 mV. The measurement was taken in the configuration of stainless steel (SS) blocking electrode/polymer electrolyte/SS electrode at ambient temperatures.

#### 2.3.2. Attenuated Total Reflectance—Fourier Transform Infrared (ATR-FTIR)

ATR-FTIR analysis was performed by Themoscientific Nicolet iS10 FTIR Spectrometer (Thermo Fisher Scientific, Waltham, MA, USA). This spectrometer is equipped with an ATR internal reflection system. The FTIR spectra were recorded with a resolution of 1 cm^−1^ in transmittance mode over the wavenumber range from 4000 and 650 cm^−1^. The FTIR spectra and peak deconvolution were scrutinized using OMNIC 8 software which is provided by Thermo Fischer Scientific Inc. The transmittance mode of FTIR spectra was initially converted into absorbance mode for peak deconvolution process. In order to deconvolute the FTIR spectra, baseline correction and curve fitting must be implemented. The FTIR curve was fitted with Gaussian–lorentzian mixed mode.

#### 2.3.3. Morphological Studies

The surface morphology of the polymer electrolytes was studied using field emission scanning electron microscope (FESEM; JEOL JSM-7600F, Akishima, Tokyo, Japan). The polymer electrolytes was coated with platinum coating to prevent charging. The images were taken at an accelerating voltage of 5 kV.

#### 2.3.4. Linear Sweep Voltammetry (LSV)

CHI600D electrochemical analyzer was used to evaluate LSV responses of filler-free polymer electrolyte and the most conducting filler-added polymer electrolyte. These cells were analyzed at a scan rate of 10 mV·s^−1^ by placing the polymer electrolyte between SS electrodes in the potential range of ±3 V.

### 2.4. Electrode Preparation

Activated carbon-based EDLC electrodes were prepared by a dip coating technique. The preparation of carbon slurry was prepared by mixing 80 wt % activated carbon (Kuraray Chemical Co Ltd., Osaka City, Japan) of particle size between 5 and 20 µm, surface area between 1800 and 2000 m^2^·g^−1^, 5 wt % carbon black (Super P), 5 wt % multi-walled carbon nanotubes (CNTs) (Sigma-Aldrich) with outer diameter, O.D. between 7 and 15 nm and length, *L* ranging from 0.5 to 10 μm) and 10 wt % poly(vinylidene fluoride) (PVDF) binder (molecular weight of 534,000 g·mol^−1^ from Aldrich) and dissolving them in 1-methyl-2-pyrrolidone(Purity≥99.5% from Merck, Darmstadt, Germany). Activated carbon was initially treated with sodium hydroxide (NaOH) and sulfuric acid (H_2_SO_4_) to increase the porosity of carbon. This slurry was stirred thoroughly for several hours at ambient temperature. The carbon slurry was then dip coated on an aluminum mesh current collector. The coated electrodes were dried in an oven at 110 °C.

### 2.5. EDLC Fabrication

EDLC cell was constructed in the configuration of electrode/polymer electrolyte/electrode. The EDLC cell configuration was eventually placed in a cell kit for further electrochemical analyses.

### 2.6. EDLC Characterization

The fabricated EDLC cell was subsequently subjected to cyclic voltammetry (CV) and galvanostatic charge-discharge (GCD) for characterization.

#### 2.6.1. Cyclic Voltammetry (CV)

The CV study of EDLC was investigated using CHI600D electrochemical analyzer (CH Instruments, Austin, TA, USA). The cell was rested for 2 s prior to the measurement. The EDLC cell was then evaluated at 10 mV·s^−1^ scan rate in the potential range between 0 and 1 V in intervals of 0.001 V. The specific capacitance (*C*_sp_) of EDLC was computed using the following equations:

(1) $$C_{\text{sp}}=\frac{i}{sm}(F\cdot g^{-1})$$

(2) $$C_{\text{sp}}=\frac{i}{sA}(F\cdot {cm}^{-2})$$

where *i* is the average anodic-cathodic current (A), *s* is the potential scan rate (V·s^−1^), *m* refers to the average mass of active materials and *A* represents surface area of the electrodes. The average mass of electrode materials is around 0.01 g, whereas the surface area of the electrode is 1 cm^−2^.

#### 2.6.2. Galvanostatic Charge-Discharge Performance (GCD)

The charge-discharge study was carried out using a Neware battery cycler. EDLC was charged and discharged at a current of 1 mA and current density of 1 mA·cm^−2^. EDLC was allowed to rest for 10 min before taking the measurements. The specific discharge capacitance (*C*_sp_) was obtained from charge-discharge curves, according to the following relationship:

(3) Csp=Im(dVdt)

where *I* is the applied current (A), *m* is the average mass of electrode materials, d*V* represents the potential change of a discharging process excluding the internal resistance drop occurring at the beginning of the cell discharge and d*t* is the time interval of discharging process. The d*V*/d*t* is determined from the slope of the discharge curve.

Energy density, *E* (W·h·kg^−1^), power density, *P* (kW·kg^−1^) and Coulombic efficiency, η (%) were assessed using the equations below

(4) $$E=\frac{C_{\text{sp}}\times {\left(dV\right)}^{2}}{2}\times \frac{1000}{3600}$$

(5) $$P=\frac{I\times dV}{2\times m}\times 1000$$

(6) $$\eta =\frac{t_{d}}{t_{c}}\times 100\%$$

where *t*_d_ and *t*_c_ are the discharging and charging times, respectively.

## 3. Results

### 3.1. Ambient Temperature-Ionic Conductivity Studies

Table 1 illustrates the ionic conductivity of polymer electrolyte membranes *versus* the weight percentage of TiO_2_.

### 3.2. Attenuated Total Reflectance—Fourier Transform Infrared (ATR-FTIR)

**Figure 1:**
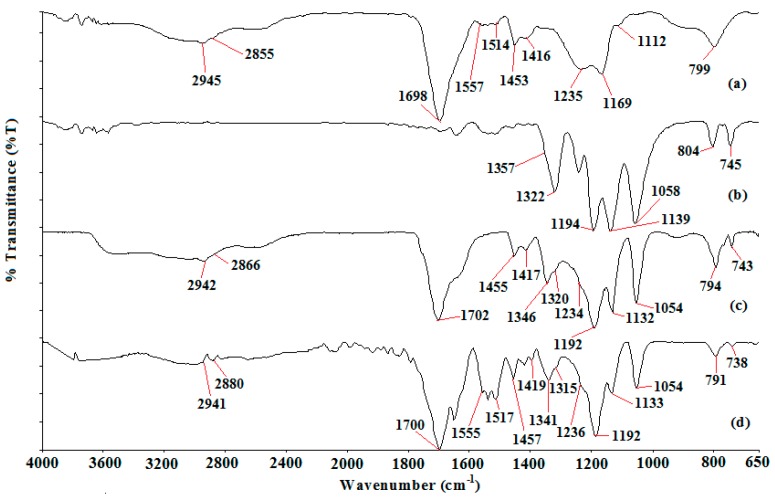
FTIR spectrum of (**a**) PAA; (**b**) LiTFSI; (**c**) Ti 0 and (**d**) Ti 3.

Figure 1 depicts the ATR-FTIR spectrum of PAA, LiTFSI, Ti 0 and Ti 3.

### 3.3. Morphological Studies

**Figure 2:**
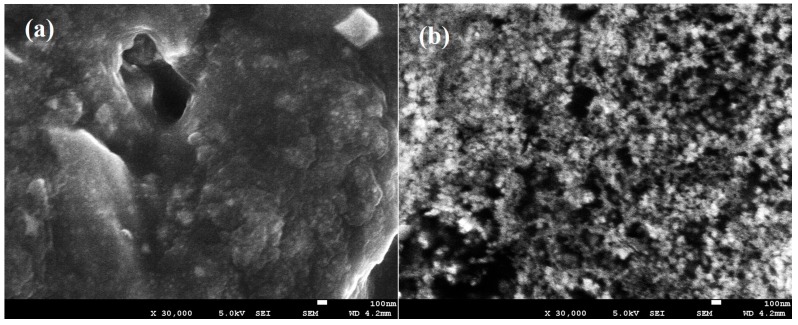
SEM images of (**a**) Ti 0 and (**b**) Ti 3.

Figure 2a,b shows the scanning electron micrograph of filler-free polymer electrolyte (Ti 0) and the highest conducting nanocomposite polymer electrolyte (Ti 3).

### 3.4. Linear Sweep Voltammetry (LSV)

**Figure 3:**
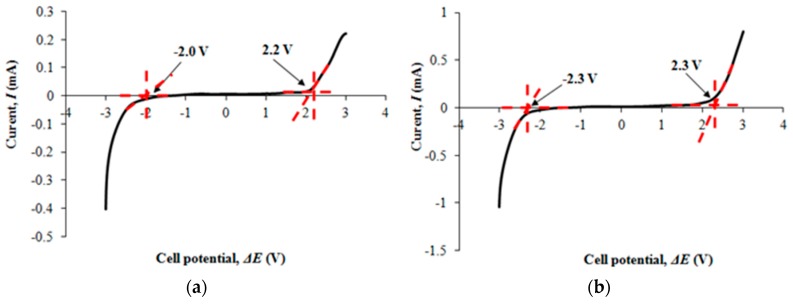
(**a**) LSV response tp filler-free polymer electrolyte; (**b**) LSV response to Ti 3.

Figure 3a,b portray the electrochemical potential window of filler-free polymer electrolyte and the highest conducting nanocomposite polymer electrolyte.

### 3.5. Cyclic Voltammetry (CV)

**Figure 4:**
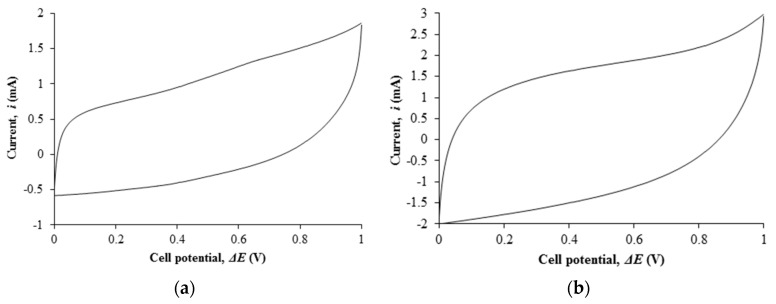
(**a**) Cyclic voltammetry of Ti 0-based EDLC; (**b**) Cyclic voltammetry of Ti 3–based EDLC.

Figure 4a,b depict the cyclic voltammetry of EDLC comprising of filler free polymer electrolyte and the highest conducting nanocomposite polymer electrolyte.

### 3.6. Galvanostatic Charge-Discharge Performance (GCD)

**Figure 5:**
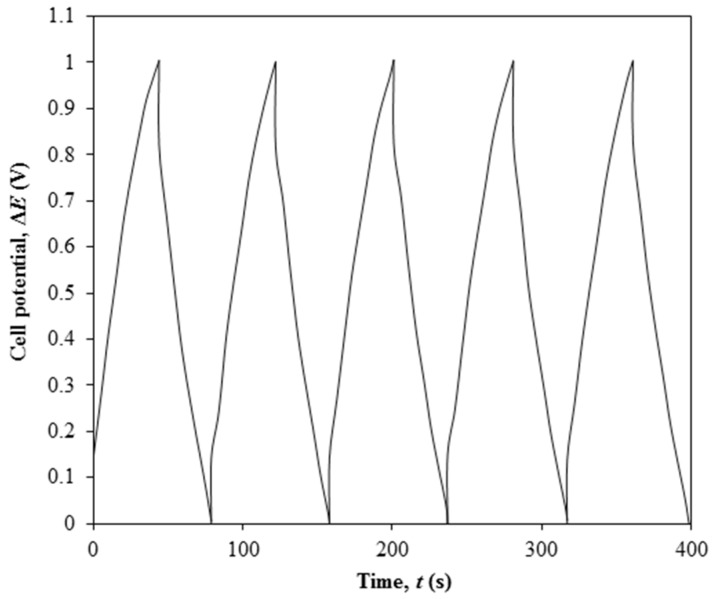
Galvanostatic charge-discharge result of Ti 3-based EDLC upon 5 cycles of charging and discharging.

Figure 5 illustrates the GCD performance of EDLC containing the highest conducting polymer electrolyte over first five cycles of charging and discharging.

**Figure 6:**
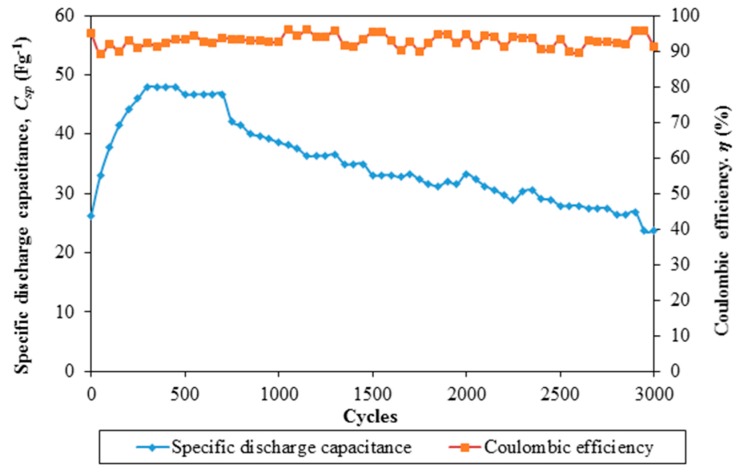
Specific discharge capacitance and Coulombic efficiency of Ti 3-based EDLC over 3000 cycles of charging and discharging.

**Figure 7:**
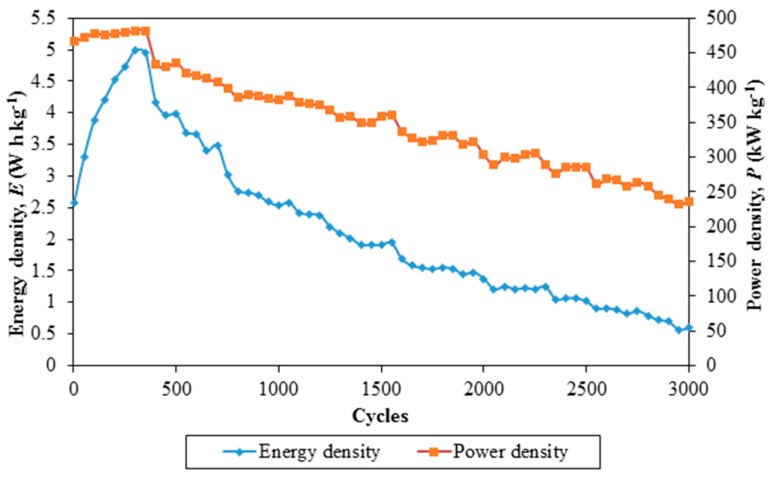
Energy density and power density of Ti 3-based EDLC over 3000 cycles of charging and discharging.

The findings on the electrochemical stability of the EDLC are illustrated in Figure 6 and Figure 7. Figure 6 exemplifies specific capacitance and Coulombic efficiency of the EDLC over 3000 cycles of charging and discharging.

In contrast, Figure 7 demonstrates energy density and power density of the EDLC over 3000 cycles of charging and discharging.

## 4. Discussion

### 4.1. Ambient Temperature-Ionic Conductivity Studies

The ionic conductivity of polymer electrolyte membranes is increased by two orders of magnitude from (1.04 ± 0.01) × 10^−6^ to (8.36 ± 0.01) × 10^−4^ S·cm^−1^ upon addition of 6 wt % of TiO_2_. Filler plays an important role as a solid plasticizer. The filler absorbs and retains the electrolyte solution in the polymer network. As a result, this retention raises the mobility of charge carriers which are transported from one site to another vacant site. The increase in ionic conductivity of nanocomposite polymer electrolyte membranes is also due to the formation of 3-D networks. Native hydroxyl groups will be formed on the surface of the filler particles when the fillers are dispersed in water. These 3-D networks can provide a new pathway (a space charge layer) for an ion conduction mechanism when the nanoparticles get closer to each other. Therefore, the ionic conductivity of nanocomposite polymer electrolyte membranes is higher than filler-free polymer electrolyte. Addition of fillers reduces the degree of crystallinity of polymer electrolyte membranes and favors the ion hopping process in the amorphous region of the polymer matrix.

**Figure 8:**
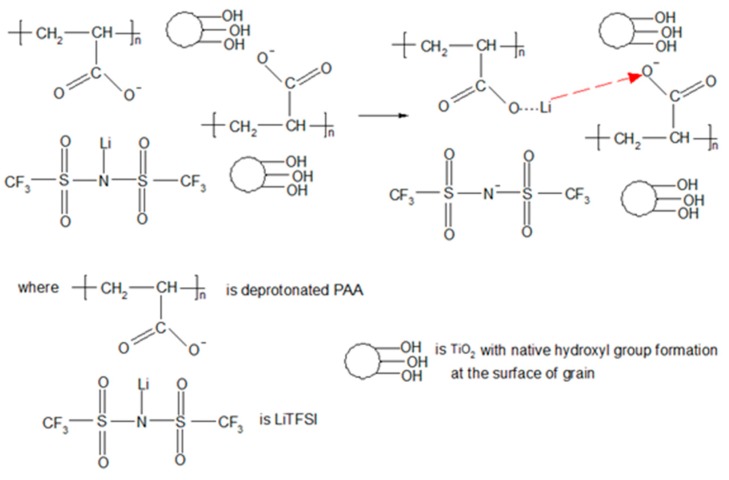
The proposed ion conduction mechanism in nanocomposite polymer electrolyte membranes.

We postulate an idea for the ion conduction mechanism in the polymer electrolyte membranes as shown in Figure 8.

We suggest that the hydrogen from the carboxylic group in PAA is initially deprotonated. As a result, carboxylate anions are produced in PAA chains after the deprotonation. Lithium cations are thus dissociated from TFSI anions due to their low lattice energy and low tendency to form ion pairs [38]. Then, the mobile lithium cations (also known as charge carriers) form the partial bond with the carboxylate anions. After that, the charge carriers are transported to another vacant site after the partial bonding between oxygen and lithium is broken down. The ionic conductivity of polymer electrolyte membranes is generated with the repeating ion transportation within the membrane. The ionic conductivity of nanocomposite polymer electrolyte is decreased gradually above addition of 6 wt % of TiO_2_. The reduction of ionic conductivity is primarily attributed to excess nano-sized particles. The aggregation of these particles would block the conducting path and impede the ion conduction in the electrolyte.

### 4.2. Attenuated Total Reflectance—Fourier Transform Infrared (ATR-FTIR)

The assignment of all possible peaks is listed in Table 2.

Ti 0 and Ti 3 exhibit all the possible bonding modes of PAA and LiTFSI, except two stretching modes. These two stretching modes are C–CH_2_ at a wave number of 1112 cm^−1^ and –(C–O)H at a wave number of 1169 cm^−1^ from PAA. The disappearance of –(C–O)H stretching mode proves the initial deprotonation of PAA as suggested in the ion conduction mechanism that we proposed in the previous section. LiTFSI spectrum manifests two weak peaks at 745 and 804 cm^−1^. The first peak is S–N stretching mode, whereas the latter peak is a combination of C–S stretching and S–N asymmetric stretching mode. The first peak exhibits a downward shift to 743 and 738 cm^−1^. The downward shift reveals the uncoordinated or free TFSI anion [43]. This latter peak becomes more intense (for Ti 0) and broader (for Ti 3) upon inclusion of PAA and TiO_2_. This peak is also shifted to 794 and 791 cm^−1^ for Ti 0 and Ti 3, respectively. These observations are mainly due to the overlapping with the weak peak at 799 cm^−1^ which is designated as the CH_2_ rocking mode of PAA. Broad band is also attained in the high wavenumber region (>2800 cm^−1^). The sp^3^ C–H stretching mode of PAA shows two broad band regions at 2855 and 2945 cm^−1^ for PAA, 2866 and 2942 cm^−1^ for Ti 0 and 2880 and 2941 cm^−1^ for Ti 3. The abrupt upward shift for the first band and downward shift for second band indicate the interaction between C–H from PAA backbone (where the carboxylate anions are connected) and Li cations.

**Figure 9:**
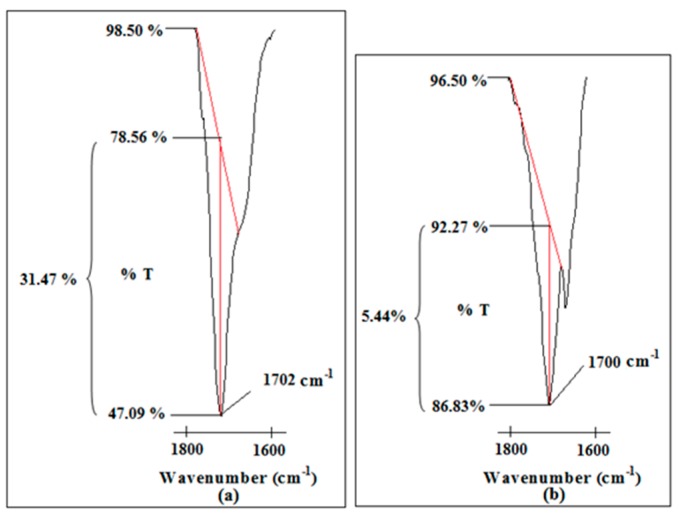
The change in peak intensity of C=O stretching mode of carboxylic group of PAA in (**a**) Ti 0 and (**b**) Ti 3.

Two weak peaks 1517 and 1555 cm^−1^ are obtained in Ti 3 compared with Ti 0. These peaks originate from C=O bending mode of PAA at 1514 cm^−1^ and –COO^−^ asymmetric stretching mode of PAA at 1557 cm^−1^ as shown in Figure 1a. The presence of the –COO^−^ asymmetric stretching mode states the deprotonation of the carboxylic acid group in PAA for ion transportation. This is further supported by the peak at 1702 cm^−1^ for Ti 0 and 1700 cm^−1^ for Ti 3. This sharp peak is assigned as C=O stretching mode of carboxylic group in PAA. The intensity of this peak is reduced greatly from 31.47% to 5.44%, in transmittance mode upon dispersion of nano-sized TiO_2_ as illustrated in Figure 9.

The reduced peak intensity infers the interaction between the carboxylate anions and lithium cations. A sharp peak, namely the S–N–S asymmetric stretching mode of LiTFSI, is obtained at 1058 cm^−1^ in the LiTFSI spectrum. This peak undergoes a downward shift to 1058 cm^−1^ for Ti 0 upon addition of PAA. The peak remains unchanged with inclusion of TiO_2_. Although the peak remains constant, the peak intensity is reduced from 38.06% to 4.31%, in transmittance mode. This verifies the effect of adding TiO_2_ in the polymer complexes.

The bands in the wavenumber region of 1400–1100 cm^−1^ were explored to further investigate the free ions, ion pairs or ion aggregation [42]. In addition, PAA demonstrates a weak peak at 1453 cm^−1^ with a shoulder peak at 1416 cm^−1^. The weak peak is assigned as the –COO^−^ group of PAA, whereas the shoulder peak is related to CH_2_ bonding mode of PAA. The shape is changed from a weak peak with a shoulder peak to two weak peaks upon addition of LiTFSI and TiO_2_. The first weak peak is located at 1455 cm^−1^ for Ti 0 and 1457 cm^−1^ for Ti 3. On the contrary, another weak peak is placed at 1417 cm^−1^ for Ti 0 and 1419 cm^−1^ for Ti 3. Apart from the insignificant change in peak location, these peaks exhibit gradual change in peak intensity. The intensity of the weak peak is decreased from 9.63% to 1.94% in transmittance mode; meanwhile, the intensity of the shoulder peak is reduced from 3.66% to 0.67% in transmittance mode. The ame peak shape is also attained in the wavenumber region of 1400–1300 cm^−1^. LiTFSI spectrum exemplifies a medium peak at 1322 cm^−1^ (assigned as S=O asymmetric stretching mode of LiTFSI) and a shoulder peak at 1357 cm^−1^ (attributed to SO_2_ asymmetric stretching mode of LiTFSI). Addition of PAA and TiO_2_ reveals an obvious change in peak shape as shown in Figure 1. The medium peak becomes a shoulder peak, whereas the shoulder peak becomes a weak peak. We notice that the weak peak is located at 1346 cm^−1^ in transmittance mode for Ti 0. This peak is shifted downwardly to 1341 cm^−1^ for Ti 3. The sign of peak shifting to a lower wavenumber indicates the presence of mobile TFSI anions in Ti 0 and Ti 3. The lower the wavenumber, the higher is the amount of mobile ions. Therefore, Ti 3 has more free TFSI anions than Ti 0.

**Figure 10:**
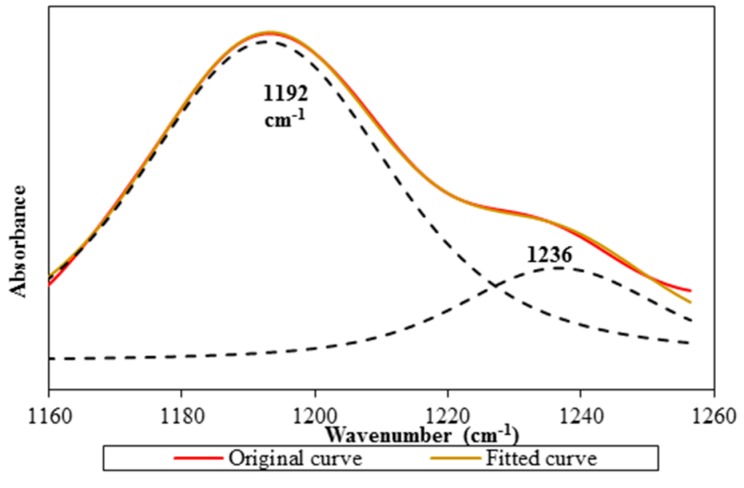
The original FTIR spectrum along with the fitted curve and deconvoluted peaks in the wavenumber region 1160–1260 cm^−1^.

LiTFSI spectrum displays two peaks at 1139 and 1194 cm^−1^ within the range between 1200 and 1100 cm^−1^. Combination of C–F stretching mode and C–SO_2_–N bonding mode of LiTFSI is the assignment for the first peak. The second peak is appointed as CF_3_ symmetric stretching mode of LiTFSI. These two peaks are still available in Ti 0 and Ti 3 spectra, but they manifest peak shifting. The first peak is shifted to 1132 cm^−1^ for Ti 0 and 1133 cm^−1^ for Ti 3, whereas the latter peak is located at 1192 cm^−1^ for both samples. An additional shoulder peak is observed in Ti 0 and Ti 3 spectra. We suggest that the presence of the shoulder peak is due to the overlapping with the peak at 1235 cm^−1^ as proven in Figure 1a. This shoulder peak is designated as C–O stretching coupled with O–H in-plane bending mode of PAA. We employ a deconvolution technique to confirm the presence of the shoulder peak as illustrated in Figure 10.

**Figure 11:**
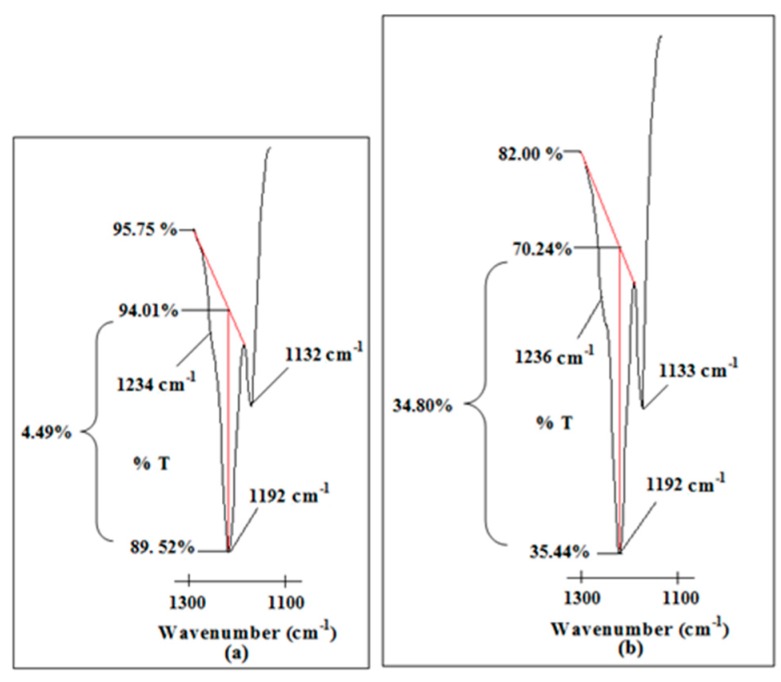
The changes in peak intensity of CF_3_ symmetric stretching mode of LiTFSI in (**a**) Ti 0 and (**b**) Ti 3.

It is noteworthy that two peaks are found at 1192 and 1236 cm^−1^ within the region. CF_3_ symmetric stretching mode is another vibrational mode to support the existence of the mobile ions. Figure 11 portrays an abrupt increase in peak intensity of CF_3_ symmetric stretching mode where the peak is increased by about 30.31% (from 4.49% to 34.80%) in transmittance mode.

The intense peak reveals that more TFSI free anions are produced in Ti 3 compared to Ti 0. As a result, these free ions could form complexation with the polymer matrix. Based on all of these findings, we can conclude that there is complexation between PAA and LiTFSI. We also prove that:

- The deprotonation of the carboxylic group in PAA has taken place for the conduction mechanism;
- Ti 3 has more mobile TFSI anions than Ti 0 which leads to higher ionic conductivity.

### 4.3. Morphological Studies

Spherulite is a highly complex ordered structure which consists of radially developed branching lamellar crystals which only normally can be observed in a material with high crystallinity. As observed in Figure 2a,b, no spherulitic structure can be observed in the SEM image of the filler-free and filler-added polymer electrolytes. This indicates the high amorphous nature of the developed polymer electrolyte samples [47,48].

The filler-free polymer electrolytes showed a considerably smooth structure as seen in Figure 2a. This confirms the complete dissolution of the LiTFSI salt in the polymer matrix as there is no separate salt phases that can be seen in the SEM image. The morphology of the polymer electrolyte was observed to be changed after the addition of the TiO_2_ filler to the polymer electrolyte. As seen in Figure 2b, the filler-added polymer electrolyte was found to have a highly porous structure (dark region) and formed a lot of random distribution of pores compared to the filler-free polymer electrolyte. The changes in this morphology is an indication of the interaction between the TiO_2_ and the polymer electrolytes [49]. The addition of the TiO_2_ filler was also found to be able to promote the formation of pores on these PAA-based polymer electrolytes. Essentially, the ionic conductivity of a solid polymer electrolyte is attainable through continuous pathways which are interconnected by pores of the membranes. Thus, a highly porous structure is very favorable for a good ion-conducting polymer electrolyte. This is in agreement with the ionic conductivities studies where the filler-added polymer electrolyte is showing higher ionic conductivities in comparison with the filler-free polymer electrolyte [50].

### 4.4. Linear Sweep Voltammetry (LSV)

The operational voltage range of filler-free polymer electrolyte is 4.2 V, ranging from −2 to 2.2 V. On the other hand, the nanocomposite polymer electrolyte shows wider potential window of 4.6 V that is from −2.3 to 2.3 V in comparison to filler-free polymer electrolyte. We observe that the potential window of polymer electrolyte is expanded upon addition of nano-sized TiO_2_. Therefore, we can conclude that addition of nano-sized fillers can improve the electrochemical properties of the electrolyte.

### 4.5. Cyclic Voltammetry (CV)

The cyclic voltammetry of Ti 0-based EDLC shows a non-ideal rectangular shape with specific capacitance of 13.46 F·g^−1^ (or equivalent to 15 mF·cm^−2^). Upon dispersion of nano-scaled fillers, the shape of the cyclic voltammetry approaches the ideal rectangular shape. The specific capacitance of EDLC containing the most conducting polymer electrolyte has doubled up to 28.56 F·g^−1^ (or equivalent to 29.54 mF·cm^−2^). The energy storage of an EDLC arises from the charge accumulation between the electrode and electrolyte interface. The transported ions in the electrolyte will form an electrical double layer on the porous surface of the carbon-based electrode. The increase in capacitance of EDLC based on the nanocomposite polymer electrolyte is due to the increase in ionic conductivity of the polymer electrolyte. The rapid ion conduction mechanism in the electrolytes leads to the rapid ion adsorption at the boundary and, thereby, increases the energy storage in the EDLC.

Surprisingly, the capacitance of EDLC assembled in this research is higher than EDLC fabricating using ionic liquid-added polymer electrolyte membranes. EDLC comprising of ionic liquid-incorporated poly(ethylene oxide) polymer electrolyte membranes and multi-walled carbon nanotubes electrodes was prepared by Pandey and co-workers [51]. The fabricated EDLC showed the specific capacitance of 2.6–3 F·g^−1^ which is 10 times lower than our current work. The capacitance that we obtained from our published work is also lower than this current work. Ionic liquid-based poly(vinyl alcohol) (PVA) polymer electrolyte membranes are studied and investigated in our previous work. The EDLC fabricated using the same electrode depicted the specific capacitance of 21.89 F·g^−1^ which is slightly lower than this work [52]. Nanocomposite polymer electrolyte-based EDLCs are also prepared and investigated by our peers. Similar work composing of PVA-lithium perchlorate (LiClO_4_)-TiO_2_ polymer electrolyte is also prepared by Lim *et al.* [53]. The specific capacitance of 12.5 F·g^−1^ was obtained in the fabricated EDLC using nanocomposite polymer electrolyte. Biopolymer electrolyte membranes based on corn starch, LiClO_4_ and nano-sized silica are prepared by Teoh [54]. However, the specific capacitance of EDLC obtained from this work is lower than our current work, which is 8.71 F·g^−1^. Therefore, we conclude that the nanocomposite polymer electrolyte is a great candidate as an electrolyte in the EDLC.

### 4.6. Galvanostatic Charge-Discharge Performance (GCD)

EDLC is charged from 0 to 1 V and then discharged from 1 to 0 V. EDLC produces the specific discharge capacitance of 26.23 F·g^−1^, energy density of 2.57 W·h·kg^−1^ and energy density of 467 kW·kg^−1^. The GCD curve shows the symmetrical shape during the charging and discharging processes with a Coulombic efficiency of 95%.

Electrochemical stability of an EDLC is the main feature if it can be applied in the devices. The cell is charged and discharged for 3000 cycles. It is noteworthy that the specific capacitance of the cell increases with the cycle number up to 47.94 F·g^−1^ in the 300th cycle. The specific capacitance of the cell remains unchanged until the 450th cycle. Beyond this cycle number, the specific capacitance of the EDLC is decreased. The specific capacitance of the cell is 23.66 F·g^−1^ at the 3000th cycle. The reduction of capacitance is suggestive of the electrolyte depletion which affects the ion transportation within the polymer matrix. On the other hand, the Coulombic efficiency of the EDLC is maintained above 90% over 3000 cycles. This infers that the nanocomposite polymer electrolyte-based EDLC has stable electrochemical properties.

Energy density and power density of the EDLC demonstrate the same trend. An abrupt increase in energy density and a gradual increase in power density are observed from the 1st cycle to the 300th cycle. This is followed up by a moderate decrease in both densities above the 300th cycle of charging and discharging. The initial energy and power densities are 2.57 W·h·kg^−1^ and 467 kW·kg^−1^, respectively. The energy density is increased to 4.94 W·h·kg^−1^ at the 300th cycle, whereas power density is increased to 481 kW·kg^−1^ at 350th cycle. The respective energy and power densities are further decreased to 0.59 W·h·kg^−1^ and 236 kW·kg^−1^ at the 3000th cycle. Nanocomposite polymer electrolyte membranes can be a good replacement for ionic liquid-based polymer electrolyte membranes. Based on these findings, EDLC comprising nanocomposite polymer electrolytes can be applied to be an energy storage device as a power system in our daily life.

## 5. Conclusions

PAA-based nanocomposite polymer electrolyte membranes are prepared and investigated in this work. Upon dispersion of nano-sized TiO_2_, the ionic conductivity of polymer electrolyte membranes is increased greatly. The complexation between PAA and LiTFSI is confirmed in FTIR analysis. SEM studies reveal that the filler-added polymer electrolytes have higher porosity. EDLC comprising nanocomposite polymer electrolytes and carbon electrodes is fabricated and studied through CV and GCD techniques. Addition of filler also increases the specific capacitance of EDLC. The fabricated EDLC shows the specific capacitance of 28.56 and 26.23 F·g^−1^ in CV and GCD curves, respectively. The maximum specific capacitance of 47.94 F·g^−1^ is achieved after charging and discharging for 300 cycles. EDLC illustrates superior Coulombic efficiency which remains above 90% over 3000 cycles. EDLC produces an energy density of 2.57 W·h·kg^−1^ and power density of 467 kW·kg^−1^. Nanocomposite polymer electrolyte is a superior electrolyte to be applied in EDLC application as it shows excellent electrochemical performance.

## Figures and Tables

**Table 1:** The composition of each material used in the polymer electrolyte membrane preparation, the designation of the polymer electrolyte membranes and the ionic conductivity of the polymer electrolyte membranes.

Sample designation	Weight percent of material (wt %)			Ionic conductivity (S·cm^−1^)
	PAA	LiTFSI	TiO_2_	
Ti 0	70	30	0	(1.04 ± 0.01) × 10^−6^
Ti 1	68.6	29.4	2	(2.88 ± 0.02) × 10^−4^
Ti 2	67.2	28.8	4	(4.04 ± 0.01) × 10^−4^
Ti 3	65.8	28.2	6	(8.36 ± 0.01) × 10^−4^
Ti 4	64.4	27.6	8	(4.65 ± 0.01) × 10^−4^
Ti 5	63	27	10	(3.22 ± 0.01) × 10^−4^

**Table 2:** Peak assignments of PAA, LiTFSI, Ti 0 and Ti 3.

Peak/band assignments	Wavenumber (cm^−1^)				References
	PAA	LiTFSI	Ti 0	Ti 3	
S–N stretching mode of LiTFSI	–	745	743	738	[39]
Combination of C–S stretching and S–N asymmetric stretching mode of LiTFSI	–	804	794	791	[39]
CH_2_ rocking mode of PAA	799	–	794	791	[40]
S–N–S asymmetric stretching mode of LiTFSI	–	1,058	1,054	1,054	[39]
C–CH_2_ stretching mode of PAA	1,112	–	–	–	[24]
Combination of C–F stretching mode and C–SO_2_ –N bonding mode of LiTFSI	–	1,139	1,132	1,133	[29,41]
–(C–O)H stretching mode of PAA	1,169	–	–	–	[42]
CF_3_ symmetric stretching mode of LiTFSI	–	1,194	1,192	1,192	[39]
C–O stretching coupled with O–H in-plane bending mode of PAA	1,235	–	1,234	1,236	[24,40]
S=O asymmetric stretching mode of LiTFSI	–	1,322	1,320	1,315	[41]
SO_2_ asymmetric stretching mode of LiTFSI	–	1,357	1,346	1,341	[39]
CH_2_ bonding mode of PAA	1,416	–	1,417	1,419	[42]
–COO^−^ group of PAA	1,453	–	1,455	1,457	[24]
C=O bending mode of PAA	1,514	–	–	1,517	[43]
–COO^−^ asymmetric stretching mode of PAA	1,557	–	–	1,555	[43,44]
C=O stretching mode of carboxylic group in PAA	1,698	–	1,702	1,700	[18,24,45,46]
sp^3^ C–H stretching mode of PAA	2,855 and 2,945	–	2,866 and 2,942	2,880 and 2,941	[24,39,42,45,46]
